# The association between cervical sagittal balance parameters and clinical outcomes after single-level surgery for cervical radiculopathy and/or stenosis: A systematic review and meta-analysis

**DOI:** 10.1016/j.bas.2026.106009

**Published:** 2026-03-24

**Authors:** Azra Gül, Javier Gomez Farias, Caroline MW. Goedmakers, Rania A. Mekary, Carmen Vleggeert-Lankamp

**Affiliations:** a Computational Neuroscience Outcomes Center, Department of Neurosurgery, Brigham and Women's Hospital, Harvard Medical School, Boston, MA, USA; b Department of Neurosurgery, Leiden University Medical Center, Leiden, the Netherlands; c Harvard T. H. Chan School of Public Health, Boston, MA, USA; d Department of Neurosurgery, Boston Medical Center, Boston, MA, USA; e School of Pharmacy, Massachusetts College of Pharmacy and Health Sciences University, Boston, MA, USA; f Department of Biostatistics, Harvard T.H. Chan School of Public Health, Boston, MA, USA; g Department of Neurosurgery, Spaarne Gasthuis, Haarlem/Hoofddorp, the Netherlands

**Keywords:** ACDF, Cervical sagittal balance, Clinical outcomes, Cobb's angle, Cervical radiculopathy

## Abstract

**Introduction:**

The relationship between cervical sagittal balance parameters and clinical outcomes after single-level anterior cervical discectomy and fusion (ACDF) for cervical radiculopathy and/or stenosis remains controversial.

**Research question:**

This meta-analysis was conducted to assess the evidence.

**Material and methods:**

Seven databases were searched through August 2025 for longitudinal studies that reported the correlation between sagittal balance and clinical outcomes. Stratified meta-analyses were conducted for the different sagittal balance parameters and clinical outcomes, accounting for the timing of assessment (preoperative, postoperative, or change scores) using the random-effects models.

**Results:**

Seven studies (with 723 patients in total) out of 693 unique studies met the inclusion criteria. A greater Cobbs angle of the target level (lordosis) appeared to be associated with a lower (better) postoperative Neck Disability Index (NDI) (pooled Pearson's r = −0.13, 95% CI: 0.23 to −0.02), while correlations with of C2–C7 angle, cSVA, T1 slope and T1 slope minus C2–C7 lordosis with NDI were not significant. No significant pooled correlations were found between postoperative C2-C7 angle and Visual Analogue Scale (VAS) arm- or neck pain. Non-pooled results demonstrated that a greater pre-operative odontoid incidence was associated with less arm and neck pain (r = −0.27; p = 0.01) and a more favorable NDI (r = −0.24; p = 0.03), and that a higher C2-C7 angle was associated with a more favorable Japanese Orthopaedic Association score (r = 0.40; p = 0.02).

**Discussion and conclusion:**

A greater postoperative Cobb angle at the target level may improve outcomes after ACDF. Further studies in larger, diverse populations are needed to confirm and generalize the results.

## Highlights

• Cervical sagittal balance may influence outcomes after ACDF, but evidence is limited.
• Greater postoperative Cobb's angle at the target level is associated with lower NDI.
• Higher preoperative odontoid incidence is associated with reduced pain.

## 1 Introduction

Cervical sagittal balance parameters are quantitative radiographic measurements that characterize the alignment of the cervical spine in the sagittal plane. The most widely used parameters include the C2-C7 Cobb angle, the C2-C7 Sagittal Vertical Axis, the T1 slope and the C7 slope (Ling et al., 2018; Azimi et al., 2021). These metrics are reproducible and reliable, facilitating objective assessment of cervical posture and alignment in both healthy and symptomatic people (Pivotto et al., 2021; Oh et al., 2023).

Currently, cervical sagittal balance parameters are integral to preoperative planning for complex cervical spine surgeries, such as deformity correction and multi-level fusion. Their use enables spine surgeons to anticipate compensatory mechanisms, optimize postoperative alignment, and minimize complications such as adjacent segment disease (Patwardhan et al., 2018; Le Huec et al., 2019). These parameters are also increasingly considered in more common procedures, including single- or two-level anterior cervical discectomy and fusion (ACDF), in which preoperative sagittal alignment may influence postoperative outcomes and risk of sagittal imbalance (Scheer et al., 2013).

Interest in cervical sagittal balance parameters has grown rapidly over the past decade, as evidenced by a significant increase in related publications and a shift in research focus toward deformity, motion preservation, and personalized surgical strategies (Lee et al., 2020). This trend reflects a broader recognition of the importance of spinal alignment in optimizing patient-reported outcomes and guiding individualized treatment approaches (Jouppi et al., 2025). Ongoing research continues to refine the understanding of normative values, interrelationships between parameters, and their impact on surgical planning and clinical results.

Nevertheless, the clinical implications of cervical sagittal balance remain controversial (Okada et al., 2009), especially within single or two-level ACDF. A better understanding could aid clinicians in selecting the most appropriate treatment and influence decisions on the extent to which sagittal balance should be restored. As a result, this study sought to elucidate the association between cervical sagittal balance parameters and clinical outcomes based on the current literature.

## 2 Methods

This systematic review and meta-analysis were conducted in accordance with the 2020 Preferred Reporting Items for Systematic Reviews and Meta-Analyses (PRISMA) checklist (Moher et al., 2009), to explore potential associations between cervical sagittal balance parameters and clinical outcomes after single or two-level ACDF in patients with cervical radiculopathy or stenosis.

### 2.1 Literature search strategy

A comprehensive systematic literature search was performed in PubMed, Medline, EMBASE, Web of Science, Cochrane Library, Emcare, and Academic Search Premier to identify relevant studies published up to August 12th, 2025. Controlled search terms were utilized, focusing on the cervical spine, sagittal alignment, and clinical outcomes. Additionally, the reference lists of included articles were meticulously cross-checked to ensure no relevant literature was overlooked (Appendix A). Covidence was utilized for the management of articles and literature for the current study (Covidence).

### 2.2 Study selection

Inclusion criteria were defined to encompass any study assessing the association between cervical sagittal balance measurements and reported clinical outcomes. Studies meeting the following criteria were included: 1) the study assessed the relationship between cervical sagittal balance parameters and clinical outcomes, 2) sagittal balance parameters were quantified using an X-ray obtained in a standing or sitting position, 3) patients were between 18 and 65 years old, 4) the study included at least 10 patients, and 5) patients underwent ACDF involving one or two cervical levels.

Articles were excluded if they were not written in English, Spanish, or Dutch, and/or if they were case reports, meta-analyses, or systematic reviews. Title and abstract screening were independently performed by two authors (AG, JGF), and a third independent author (CVL) was consulted in the case of conflicting evaluations.

### 2.3 Risk of bias assessment

To identify the potential risk of bias, two authors (AG, JGF) independently evaluated the quality of each included study using the Newcastle Ottawa Scale (Wells et al., 2023). The Newcastle Ottawa Scale assesses three domains: subject selection, comparability, and assessment of outcome, with a total of nine possible points. Following this evaluation, the studies were categorized into two groups based on their risk of bias, with low risk of bias (5-9 points) and a high risk of bias (4 or fewer points) using a methodology adapted from Furlan et al. (2009). In case of any disagreements, the authors discussed and resolved them among themselves. If a consensus could not be reached, a third author provided the final judgment (CVL).

In line with the PRISMA checklist (Moher et al., 2009), small study effect analysis was not performed for any outcomes, because none included ≥10 studies, as recommended in the Cochrane Handbook (Page et al., 2024).

### 2.4 Outcome measures

For each included article, the following data were extracted: author, study design, year of publication, sample size, study quality, reported clinical outcomes, cervical sagittal balance measurements, and mean duration of follow-up time. Clinical outcomes and cervical sagittal balance parameters were variably assessed across studies, either preoperatively, postoperatively, or as change scores calculated between preoperative and postoperative measurements. Age, gender, and smoking history were also extracted.

#### 2.4.1 Clinical outcomes

The clinical outcomes Neck Disability Index (NDI), Visual Analogue scale (VAS) and the Japanese Orthopedic Association Score (JOA), including their timing of measurement, were retrieved from the eligible studies. We documented whether outcomes were reported as preoperative scores, postoperative scores, or as changes from preoperative baseline values.

*2.4.1.1 The NDI* is a questionnaire consisting of 10 scaled items that assess neck functionality through three key aspects of neck complaints: pain intensity, daily work-related activities, and non-work-related activities. Each item is rated on a scale from 0 to 5, with the total raw score ranging from 0 (indicating no disability) to 50 (indicating the highest level of disability), calculated into a 0 to 100 percent scale (Vernon and Mior, 1991).

*2.4.1.2 The VAS* arm or neck pain score illustrates the disabling pain in the arm or neck with a scoring ranging from 0 to 100 mm 0 mm means ‘no pain or tingling sensations’ and 100 mm means ‘the most terrible pain or tingling sensations I can imagine’. Reliability, validity, and responsiveness of the VAS have been demonstrated previously (Huskisson, 1974; Carlsson, 1983).

*2.4.1.3 The (modified) JOA score* is a disease-specific, physician-reported scale designed to evaluate neurological function and allows surgeons to measure pre- and postintervention changes (Dalitz and Vitzthum, 2019; Bartels et al., 2010). The scale ranges from 0, representing the most severe impairment to 17, which indicates normal neurological function without deficits.

#### 2.4.2 Sagittal balance parameters

The included papers demonstrate a variety of cervical sagittal balance parameters. Merging synonyms of cervical sagittal balance parameters allowed us to pool results.

**Fig. 1:**
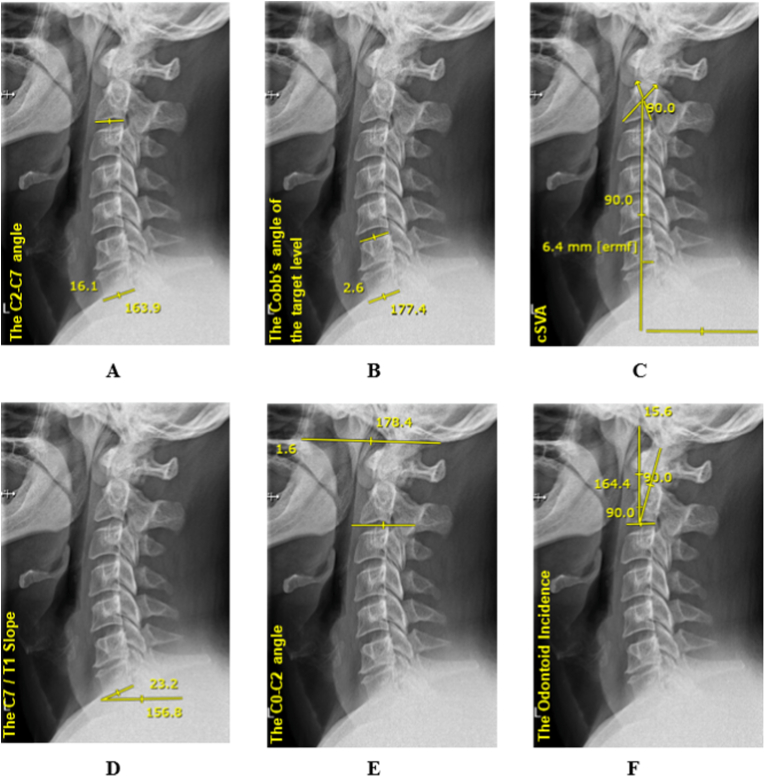
Measurement of cervical sagittal balance parameters; (a) The C2-C7 angle, (b) The Cobb's angle of the target level, (c) The cervical sagittal vertical axis, (d) The C7/T1 slope, (e) The C0-C2 angle, (f) the odontoid incidence.

*2.4.2.1 The C2-C7 angle*, also referred to as the Cobb's angle, C2-C7 lordosis, or cervical lordosis, is the angle formed by the intersection of two lines drawn along the upper endplate of the C2 vertebra and the lower endplate of the C7 vertebra (Martini et al., 2021) (Fig. 1).

*2.4.2.2 The Cobb's angle of the target level*, also referred to as ‘the segmental angle’, is measured through the angle between adjacent vertebrae in the cervical spine (Chiu et al., 2022). (Fig. 1)

*2.4.2.3 The cervical sagittal vertical axis (cSVA)*, also referred to as C2-C7 sagittal vertical axis, is the horizontal distance between a vertical reference line through the center of C2 and the superior posterior corner of C7 (Ye et al., 2020) (Fig. 1).

*2.4.2.4 The T1 slope* is defined as the angle between a line drawn parallel to the superior endplate of the T1 vertebra and a horizontal reference line. The C7 slope is defined as the angle between the horizontal line and a line drawn along the lower endplate of the C7 vertebra. As both the upper and lower C7 slopes demonstrate a strong correlation with the T1 slope, the C7 angle may serve as a reliable substitute for estimating the T1 slope when the superior endplate of T1 is not clearly visualized. Therefore, we merged the results of the C7 and T1 slopes into ‘t1 slope’ (Chen et al., 2020) (Fig. 1).

*2.4.2.5 The T1 slope minus C2-C7 lordosis (T1 slope - C2-C7 lordosis)* is a calculated parameter that helps to assess the overall spinal alignment by comparing the T1 slope with the cervical lordosis. (Chiu et al., 2022) (Fig. 1).

*2.4.2.6 The C0-C2 angle* is the angle measured between the McGregor's line and the inferior endplate line of C2. The McGregor's line connects the posterior edge of the hard palate and the most caudal point of the occipital bone (Lin et al., 2020) (Fig. 1).

*2.4.2.7 Thoracic Inlet Angle* was defined as the angle formed between a vertical line passing through the midpoint of the superior endplate of T1 and a second line extending from this midpoint to the tip of the sternum (Chen et al., 2020).

2.4.2.8 *Cranial tilt* was measured as the angle between a line drawn through the apex of the odontoid process and a second line perpendicular to the superior endplate of T1. The angle is considered positive when directed anteriorly and negative when directed posteriorly (Lin et al., 2020).

*2.4.2.9 Cervical tilt* was defined as the angle between a line perpendicular to the superior endplate of T1 and a line extending from the apex of the odontoid process. Anteriorly directed angles were labelled positive, while posteriorly directed angles were considered negative (Lin et al., 2020).

*2.4.2.10 The odontoid incidence (OI)* was defined as the angle between a line drawn perpendicular to the midpoint of the C2 endplate and a line extending from this point to the center of the odontoid process (Gökoğlu et al., 2025; Gong et al., 2025).

### 2.5 Statistical analysis

The studies reported the Pearson correlation coefficients to assess the association between cervical sagittal balance parameters and the clinical outcomes. Data collection was performed by two authors (AG, JGF), and a third author solved any discrepancies (CVL). The timing of the clinical outcome and the timing of the sagittal balance parameters were important for the subgroup analysis. Moreover, the effect of direction was collected. All of the pooled point estimates for the reported correlation coefficients and their respective 95% confidence intervals were calculated using a random effects model (DerSimonian and Laird, 1986). Forest plots were used to visualize individual and summary estimates. The Higgins I^2^ index was used to evaluate heterogeneity, an I^2^ value of more than 50% was considered high (Higgins et al., 2003). Comprehensive Meta-Analysis version 4 was utilized to perform statistical analysis.

## 3 Results

### 3.1 Literature search

**Fig. 2:**
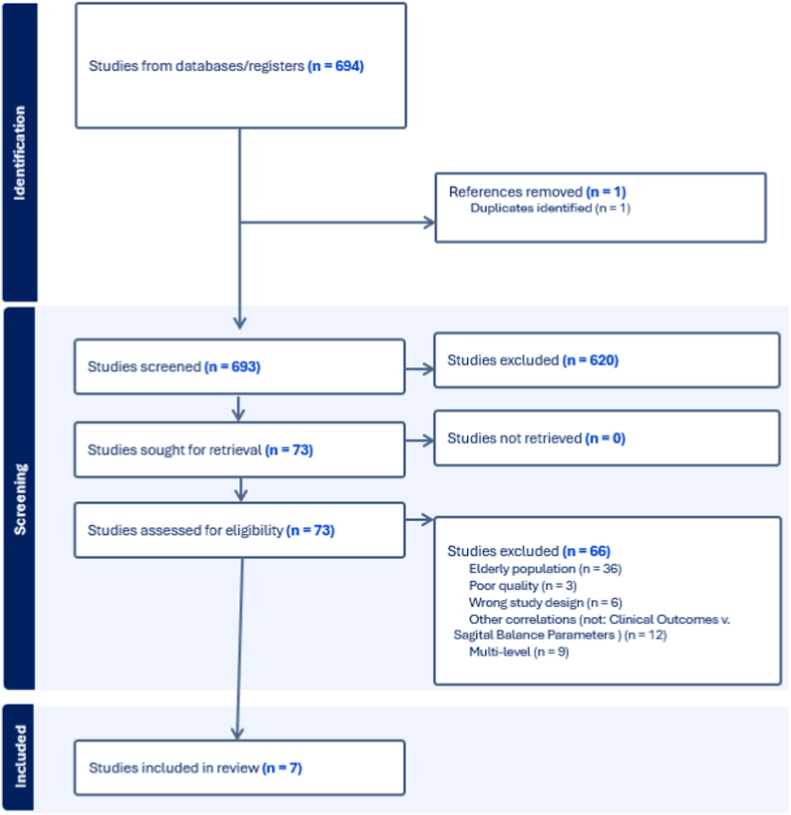
Flow Diagram-Studies selection progress.

There were 694 articles identified from PubMed, Medline, EMBASE, Web of Science, Cochrane Library, Emcare and Academic Search Premier, representing 693 unique studies. After title and abstract screening, 73 articles were found to be eligible for full-text screening, and subsequent assessment yielded 7 relevant articles that were included in this study (Chen et al., 2020; Lin et al., 2020; Wang et al., 2021; Xu et al., 2020; Ismaeil et al., 2021; Gökoğlu et al., 2025; Gong et al., 2025) (Fig. 2).

### 3.2 Demographics, clinical features, study characteristics

The sample size ranged between 30 and 212 patients, with a total of 727 patients in all seven studies combined. The pooled mean age of the patients ranged from 43.6 to 52.8 years. Smoking history was assessed in one study (Lin et al., 2020), in which 14/68 patients were smokers. The follow-up ranged from 0.25 to 43.2 months (Table 1). All seven studies were placed in the low risk of bias category as defined by the adapted Furlan et al. methodology (Furlan et al., 2009; Chen et al., 2020; Lin et al., 2020; Wang et al., 2021; Xu et al., 2020; Ismaeil et al., 2021; Gökoğlu et al., 2025; Gong et al., 2025). Agreement between reviewers (AG and JGF) was high, with minor discrepancies that were resolved through discussion (Appendix B).

**Table 1:** Baseline population characteristics of patients who underwent anterior discectomy due to radiculopathy or stenosis described in the studies included in the meta-analysis.

First author, Year, country	Population and procedure (design, n)	Follow-up duration (months)	Mean age ± SD	Male: Female	Smoking (n)	Sagittal Balance Parameters Measured
Chen J, 2020, China (Chen et al., 2020)	single or two-level ACCF or ACDF (retrospective cohort, 158)	3	52.1 ± 9.2	96:62		C2-C7 angle^a^,^b^ t1 slope^a^,^b^ thoracic inlet angle^a^,^b^
Lin Z, 2020, China (Lin et al., 2020)	Radiculopathy, single-level ACDF (retrospective cohort, 68)	0.25	48.8 ± 9.0	39:29	14	Cobb's angle Target Level^c^ t1 Slope^c^ C2-C7 angle^c^ cervical tilt^c^ cranial tilt^c^
Wang X, 2021, China (Wang et al., 2021)	Radiculopathy or myelopathy single-level ACDF (observational cohort, 132)	43.2	43.6 ± 7.6	66:66		Cobb's angle Target Level^a^ C2-C7 angle^a^ t1 slope^a^ sagittalverticalaxis^a^ t1 slope minus C2-C7 Lordosis
Xu Y, 2020, China (Xu et al., 2020)	Radiculopathy or myelopathy single-level ACDF (retrospective cohort, 212)	14.7	52.6 ± 10	147:65		C2-C7 angle^a^ t1 slope^a^ sagittalverticalaxis^a^ neck tilt^a^ thoracic inlet angle^a^
Ismaeil AS, 2020, Egypt (Ismaeil et al., 2021)	Single-level ACDF (retrospective cohort, 30)	6	44.5 ± 7.6	16:14		C2-C7 angle^a^ t1 slope^a^ sagittalverticalaxis^a^ C0-C2 angle^a^
Gökoglu A, 2025, Türkiye (Gökoğlu et al., 2025)	Single level ACDF (retrospective cohort, 36)	6	46.3 ± 12.89	14:22		C2-C7 angle^c^
Gong Y, 2025 China (Gong et al., 2025)	Myelopathy, single-level ACDF (retrospective cohort, 87)	24	52.8 ± 11.31	34:53		odontoid incidence^b^

ACCF; Anterior cervical corpectomy with fusion, ACDF; anterior cervical discectomy and fusion.

a Postoperative.

b Preoperative.

c Change score.

#### 3.2.1 Pooled measurements: postoperative VAS arm- and/or neck pain outcome

**Fig. 3:**
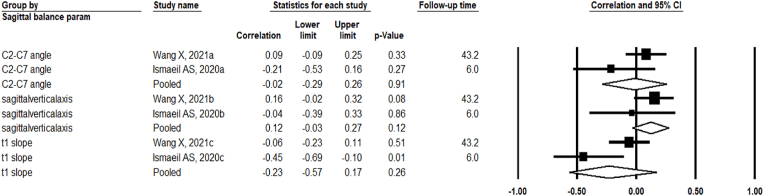
Pooled Correlations between the VAS arm- and/or neck pain compared to Cervical Sagittal Balance Parameters, both measured postoperatively.

Four articles reported VAS arm- and/or neck pain as a clinical outcome parameter (Lin et al., 2020; Wang et al., 2021; Ismaeil et al., 2021; Gong et al., 2025), of which only two provided sufficiently comparable data to be included in the pooled analysis (Fig. 3; 26, 28). The remaining two could not be pooled due to differences in measurement timing (Table 2; 25, 30). Upon stratifying by sagittal balance parameters, the pooled analysis showed no significant association between the postoperative C2-C7 angle and postoperative VAS arm- and/or neck pain (pooled r = −0.02, 95% CI: 0.29; 0.26, p = 0.91, I^2^: 50.4%). Similarly, no significant associations were found between the postoperative sagittal vertical axis and postoperative VAS arm- and/or neck pain (pooled r = 0.12, 95% CI: 0.03; 0.27, p = 0.12, I^2^ = 0%), nor between the postoperative T1 slope and postoperative VAS arm- and/or nek pain (pooled r = −0.23, 95% CI: 0.57; 0.17, p = 0.26, I^2^ = 74.8%) (Fig. 3).

**Table 2:** Non-pooled correlations.

Group by		Study Name	Statistics for Each Study		Follow-up time (months)
Clinical Outcome	Sagittal Balance Parameter		Correlation	p-value	
JOA Score^a^	C2-C7 angle^a^	Wang X, 2021a	0.02	0.80	43.2
JOA Score^c^	C2-C7 angle^b^	Chen J, 2020b	0.08	NS	3
JOA Score^c^	C2-C7 angle^a^	Chen J 2020 f	−0.03	NS	3
JOA Score^c^	C2-C7 angle^c^	Gökoglu A, 2025	0.40	0.02	6
JOA Score^a^	sagittalverticalaxis^a^	Wang X, 2021c	0.13	0.14	43.2
JOA Score^a^	Cobbs angle target level^a^	Wang X, 2021b	0.14	0.12	43.2
JOA Score^a^	T1 slope^a^	Wang X, 2021d	−0.01	0.92	43.2
JOA Score^c^	T1 slope^b^	Chen J, 2020a	0.02	NS	3
JOA Score^c^	T1 slope^a^	Chen J, 2020e	−0.12	NS	3
JOA Score^a^	T1 slope – C2-C7 lordosis^a^	Wang X, 2021e	0.06	0.47	43.2
JOA Score^a^	Odontoid Incidence^b^	Gong Y, 2025	0.12	0.27	24
JOA Score^c^	Thoracic inlet angle^b^	Chen J, 2020	−0.13	0.01	3
VAS Score^a^	C0-C2 angle^a^	Ismaeil AS, 2020	0.30	0.11	6
VAS Score^a^	Cobbs angle target level^a^	Wang X, 2021a	−0.11	0.23	43.2
VAS Score^c^	Cobbs angle target level^a^	Lin Z, 2020a	−0.11	NS	6
VAS Score^c^	T1 slope^c^	Lin Z, 2020b	0.17	NS	0.25
VAS Score^a^	T1 slope – C2-C7 Lordosis^a^	Wang X, 2021b	−0.17	0.15	43.2
VAS Score^a^	Odontoid Incidence^b^	Gong Y, 2025	−0.27	0.01	23
VAS Score^c^	Cranial tilt^c^	Lin Z, 2020	−0.17	NS	0.25
VAS Score^c^	Cervical tilt^c^	Lin Z, 2020	0.19	NS	0.25
NDI^a^	C0-C2 angle^a^	Ismaeil, AS, 2020	0.63	0.24	6
NDI^c^	Cobbs angle target level^c^	Lin Z, 2020	−0.05	NS	0.25
NDI^c^	T1 slope^c^	Lin Z, 2020	0.14	NS	0.25
NDI^a^	Odontoid Incidence^b^	Gong Y, 2025	−0.24	0.03	23
NDI^a^	Thoracic inlet angle^a^	Xu Y, 2020	0.05	NS	14.7
NDI^c^	Cranial tilt^c^	Lin Z, 2020	−0.04	NS	0.25
NDI^c^	Cervical tilt^c^	Lin Z, 2020	0.04	NS	0.25

NS: Non-significant *(paper only provided whether the correlation was significant or not, they did not provide the p-value).*

Timing of measurement.

a Postoperative.

b Preoperative.

c Change Score (difference between postoperative and preoperative measurements).

#### 3.2.2 Pooled measurements: postoperative NDI outcome

**Fig. 4:**
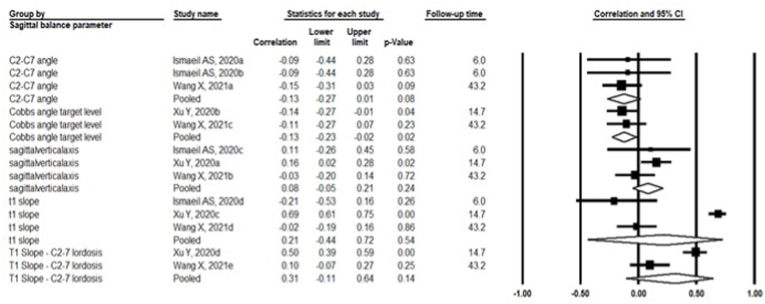
**Pooled Correlations between the NDI compared to Cervical Sagittal Balance Parameters,***both measured postoperatively*. Grote heterogeniteit bij1-t1 slope en ndi - > groot verschil in follow-up time2-mismatch t1 c2c7 en ndi.

Three studies evaluated the association between postoperative cervical sagittal balance parameters and postoperative NDI scores (Wang et al., 2021; Xu et al., 2020; Ismaeil et al., 2021). A statistically significant inverse correlation was observed between the Cobbs angle at the target level and NDI scores (pooled r = −0.13, 95% CI: 0.23; −0.02, p = 0.02, I^2^ = 0%), indicating that a greater Cobbs angle of the target level (lordosis) was associated with a lower (better) NDI score (Fig. 4). No significant association was observed between the C2-C7 angle and NDI scores (pooled r = −0.13, 95% CI: 0.27; 0.01, p = 0.08, I^2^ = 0%), even though the observed beneficial association was as strong as the one observed with the Cobbs angle of the target level.

Likewise, no significant associations were identified between the sagittal vertical axis and the NDI (pooled r = 0.08, 95% CI: 0.05; 0.21, p = 0.24, I^2^ = 28.8%), the T1 slope and NDI (pooled r = 0.21, 95% CI: 0.44; 0.72, p = 0.54, I^2^ = 97.2%) or the mismatch between T1 and C2-C7 Lordosis and the NDI (pooled r = 0.31, 95% CI: 0.11; 0.64, p = 0.14, I^2^ = 93.6%) (Fig. 4).

### 3.3 Measurements that could not be pooled

Due to heterogeneity in the cervical sagittal balance measurements across studies and the timing of outcome assessments, several correlations could not be pooled. An overview of these individual correlations was presented in Table 2.

The association between the C2-C7 angle and JOA scores was evaluated through four distinct comparisons derived from three studies (Chen et al., 2020; Wang et al., 2021; Gökoğlu et al., 2025). One study found no significant association between the postoperative C2-C7 angle and the postoperative JOA score after a mean follow-up of 43.2 months (r = 0.02, p = 0.80) (Wang et al., 2021). A second study reported non-significant correlations between preoperative C2-C7 angle and the change in JOA score (r = 0.08), as well as between the postoperative C2-C7 angle and the change in JOA score (r = −0.03) (Chen et al., 2020). In contrast, a third study reported a statistically significant positive correlation between the change in C2-C7 angle and the change in JOA score (r = 0.40, p = 0.02) (Gökoğlu et al., 2025).

The relationship between the T1 slope and JOA scores was examined using three different comparisons from two studies (Chen et al., 2020; Wang et al., 2021). When comparing postoperatively measured T1 slope with postoperative JOA score, no significant association was observed (r = −0.01, p = 0.92) (Wang et al., 2021). Similarly, no significant correlations were found between preoperative T1 slope and change in JOA score (r = 0.02), or between postoperative T1 slope and change in JOA score (r = −0.12) (Chen et al., 2020).

The pre-operative odontoid incidence was examined in relationship to three clinical outcomes (Gong et al., 2025). First, a significant negative correlation was observed between the preoperative odontoid incidence and the postoperatively assessed VAS arm, - and/or neck pain (r = −0.27, p = 0.01), indicating that a higher baseline odontoid incidence was associated with lower postoperative pain levels. Likewise, a significant negative correlation was found with the postoperative NDI (r = −0.24, p = 0.03), suggesting that greater baseline odontoid incidence corresponded with better postoperative NDI outcomes. Finally, the association between preoperative odontoid incidence and the postoperative JOA score was non-significant (r = 0.12 p = 0.27).

One article evaluated the relationship between preoperative thoracic inlet angle and change in JOA score, which demonstrated a significant negative correlation (r = −0.13, p = 0.01) (Chen et al., 2020).

Given the diversity of sagittal balance parameters and clinical outcome measures across studies, numerous additional correlations were reported; however, these are not discussed in detail here due to substantial variability in measurement timing and reporting methods.

## 4 Discussion

This systematic review comprehensively evaluated the association between cervical sagittal balance parameters and clinical outcomes in patients who underwent single-level ACDF, encompassing three distinct clinical outcomes and eleven sagittal balance parameters, including the timing of both their assessment, based on seven studies involving a total of 723 individuals. The findings suggested that a greater postoperatively measured Cobb angle at the target level was associated with reduced neck-related functional disability (NDI). In the non-pooled measurements, three significant associations were identified. Although these results should be interpreted with caution due to the limited number of studies and the observed heterogeneity, these findings highlight the potential of sagittal balance parameters to inform surgical planning, improve outcome prediction, and support tailored, patient-specific treatment strategies, while additionally providing a foundation for future research to refine individualized treatment.

Focusing on the observed relationship between the Cobb angle at the target level and the NDI, the pooled analysis included two studies, both reporting an inverse correlation. One study, comprising 212 patients, found this relation to be statistically significant (Xu et al., 2020), whereas the other study, with 132 patients, could not demonstrate a significant association (Wang et al., 2021). It is reasonable to hypothesize that a larger Cobb angle at the target cervical level is associated with a lower NDI (indicating better clinical outcomes), as increased cervical lordosis reduces compensatory muscular effort and abnormal stress on the cervical spine, thereby alleviating pain and improving function. This is additionally reflected by the non-statistically significant negative association between C2-C7 angle and the NDI. Despite the lack of statistical significance, the comparable effect size suggests that limited statistical power may underlie this finding. Other pooled analyses did not reveal significant associations between the cervical sagittal balance parameters and clinical outcomes, highlighting the limited and heterogeneous nature of the current evidence.

The measurements that could not be pooled demonstrated three significant correlations. A direct correlation between the change in C2-C7 angle and the change in JOA score, (Gökoğlu et al., 2025), indicating that larger cervical lordosis (C2-C7 angle) was associated with better clinical outcome (higher JOA score). Moreover, inverse correlations were identified between preoperative odontoid incidence and both postoperative NDI and postoperative VAS arm- and/or neck pain, suggesting that higher baseline odontoid incidence was associated with better postoperative NDI and VAS outcomes. A higher baseline odontoid incidence may reflect a more favorable intrinsic cervical alignment, reducing the need for compensatory muscular activity and thereby lowering mechanical strain. This possible biomechanical advantage could plausibly contribute to reduced pain and improved postoperative neck function. Lastly, a smaller preoperative thoracic inlet angle was associated with a larger change in the JOA score, potentially reflecting that patients with less favorable preoperative sagittal alignment had more room for improvement, or that surgical correction yielded greater functional gains in these patients (Takakura et al., 2024).

It is important to consider which cervical sagittal balance parameters are most relevant and why, to guide future research. The studies included above assessed preoperative and postoperative measurements, as well as the changes between these time points. For the C2–C7 angle, it may also be informative to evaluate the difference between flexion and extension radiographs, and to explore whether changes in this dynamic range of motion correlate with improved clinical outcomes. One could hypothesize that reduced pain would be associated with increased cervical mobility. Additionally, a larger cSVA may contribute to greater pain, as a larger SVA increases the anterior load on the cervical spine, leading to greater compensatory muscle activity and mechanical stress. However, this relationship was not observed in the current literature, which may be due to limited data or because the cSVA in these cohorts was relatively normal, i.e., not sufficiently deviated to detect clinically relevant effects.

Furthermore, the T1 slope represents a relevant sagittal parameter. While a large T1 slope is generally associated with the development of cervical lordosis, this relationship is not absolute (Tao et al., 2021). Consequently, the T1-C2-C7 mismatch may provide an informative assessment of sagittal alignment. In this review, only one study evaluated this parameter, underscoring the need for further investigation. Notably, analyses involving the T1-slope demonstrated substantial heterogeneity, which may partly be explained by differences in postoperative follow-up duration across studies. Although the T1 slope itself is considered a relatively fixed anatomical measure (Staub et al., 2018), postoperative compensatory alignment changes may influence its associations with clinical outcomes over time, while preoperative values may still serve as potential predictors of recovery. Moreover, examining the relationship between the T1 slope and the cSVA, as well as their combined association with clinical outcomes, may provide additional insight into the biomechanical determinant of recovery after ACDF.

Our meta-analysis had several limitations. First, some pooled analyses lacked statistical power due to the limited number of studies reported correlations (with p-values or 95%Cis) for the same cervical sagittal balance parameter, clinical outcome, and time point (preoperative, postoperative, or change score). Second, it should be acknowledged that most included studies (6/7) were conducted in Asian populations, which may restrict the generalizability of our findings to other ethnic groups. Consequently, the applicability of these results to non-Asian populations, such as Caucasians and Hispanics, should be interpreted with caution. These limitations underscore the need for further research with larger, more diverse cohorts to validate and expand upon the current findings. Nonetheless, several strengths are noteworthy. In order to reduce clinical and statistical heterogeneity, studies involving multi-level ACDF were excluded, as multi-level procedures often involve more complex biomechanics and variable postoperative outcomes compared to single-level interventions. Moreover, including multi-level ACDF studies could have introduced confounding factors related to differences in surgical technique, extent of fusion, and adjacent segment effects, which may obscure the specific relationship between sagittal balance parameters and clinical outcomes. Lastly, to maximize homogeneity, all of our pooled results were stratified by the different sagittal balance parameters in relation to each of the clinical outcomes.

## 5 Conclusion

The available evidence suggests that the Cobb angle at the target level, may influence clinical outcomes following single-level ACDF. Although current evidence is limited, these findings underscore the potential relevance of sagittal balance in surgical planning and individualized patient care. By delineating the key sagittal parameters of interest, this review offers a framework for future research to further elucidate these associations using standardized assessment methods in larger and more diverse patient populations.

## Funding

This research did not receive specific grants from funding agencies in the public, commercial, of not-for-profit sectors.

## Declaration of competing interest

The authors declare that they have no known competing financial interests or personal relationships that could have appeared to influence the work reported in this paper.
