# Supplementary material for: The association between cervical sagittal balance parameters and clinical outcomes after single-level surgery for cervical radiculopathy and/or stenosis: A systematic review and meta-analysis
Source: Brain Spine. 2026 Mar 24;6:106009. doi: 10.1016/j.bas.2026.106009 (PMC13087681; doi:10.1016/j.bas.2026.106009)
Supplement: Multimedia component 1 [file mmc1.docx]

**Appendix A: Search Strategy:**

**Pubmed**490 Hits on Aug 12, 2025

(("Cervical Vertebrae"[mesh] OR "cervical spine"[tw] OR "cervical spines"[tw] OR "cervical spinal"[tw] OR "Cervical Vertebrae"[tw] OR "Cervical Vertebra"[tw] OR "Cervical Atlas"[tw] OR "odontoid process"[tw] OR "Cervical Cord"[mesh] OR "Cervical Cord"[tw] OR "cervical"[tw] OR "cervical*"[tw] OR "C1 spine"[title/abstract:~6] OR "C2 spine"[title/abstract:~6] OR "C3 spine"[title/abstract:~6] OR "C4 spine"[title/abstract:~6] OR "C5 spine"[title/abstract:~6] OR "C6 spine"[title/abstract:~6] OR "C7 spine"[title/abstract:~6] OR "C1 vertebra"[title/abstract:~6] OR "C2 vertebra"[title/abstract:~6] OR "C3 vertebra"[title/abstract:~6] OR "C4 vertebra"[title/abstract:~6] OR "C5 vertebra"[title/abstract:~6] OR "C6 vertebra"[title/abstract:~6] OR "C7 vertebra"[title/abstract:~6] OR "C1 vertebral"[title/abstract:~6] OR "C2 vertebral"[title/abstract:~6] OR "C3 vertebral"[title/abstract:~6] OR "C4 vertebral"[title/abstract:~6] OR "C5 vertebral"[title/abstract:~6] OR "C6 vertebral"[title/abstract:~6] OR "C7 vertebral"[title/abstract:~6]) AND ("Spondylosis"[mesh] OR "spondylosis"[tw] OR "Spondylolysis"[tw] OR "degenerative"[tw] OR "degenerat*"[tw]) AND ("sagittal alignment"[tw] OR "sagittal alignments"[tw] OR "sagittal balance"[tw] OR "sagittal balanced"[tw] OR "sagittal balances"[tw] OR "sagittal balancing"[tw] OR "sagittal profile"[tw] OR "sagittal parameters"[tw] OR "sagittal parameter"[tw] OR "sagittal alignment"[title/abstract:~22] OR "sagittal alignments"[title/abstract:~22] OR "sagittal balance"[title/abstract:~22] OR "sagittal imbalance"[title/abstract:~22] OR "sagittal balanced"[title/abstract:~22] OR "sagittal balances"[title/abstract:~22] OR "sagittal balancing"[title/abstract:~22] OR (("Postural Balance"[Mesh] OR "Posture Balance"[tw] OR "Posture Equilibrium"[tw] OR "Musculoskeletal Equilibrium"[tw] OR "Postural Equilibrium"[tw] OR "Postural Control"[tw] OR "Posture Control"[tw]) AND ("sagittal"[tw] OR "sagittal*"[tw]))) NOT (("Case Reports"[ptyp] OR "case report"[ti] OR "case rep"[all fields]) NOT ("Review"[ptyp] OR "review"[ti] OR "Clinical Study"[ptyp] OR "trial"[ti] OR "RCT"[ti])) AND (english[la] OR dutch[la]))

**Medline**486 hits, 0 unique
((exp "Cervical Vertebrae"/ OR "cervical spine".mp OR "cervical spines".mp OR "cervical spinal".mp OR "Cervical Vertebrae".mp OR "Cervical Vertebra".mp OR "Cervical Atlas".mp OR "odontoid process".mp OR exp "Cervical Cord"/ OR "Cervical Cord".mp OR "cervical".mp OR "cervical*".mp OR (("C1" ADJ6 "spine") OR ("C2" ADJ6 "spine") OR ("C3" ADJ6 "spine") OR ("C4" ADJ6 "spine") OR ("C5" ADJ6 "spine") OR ("C6" ADJ6 "spine") OR ("C7" ADJ6 "spine") OR ("C1" ADJ6 "vertebra") OR ("C2" ADJ6 "vertebra") OR ("C3" ADJ6 "vertebra") OR ("C4" ADJ6 "vertebra") OR ("C5" ADJ6 "vertebra") OR ("C6" ADJ6 "vertebra") OR ("C7" ADJ6 "vertebra") OR ("C1" ADJ6 "vertebral") OR ("C2" ADJ6 "vertebral") OR ("C3" ADJ6 "vertebral") OR ("C4" ADJ6 "vertebral") OR ("C5" ADJ6 "vertebral") OR ("C6" ADJ6 "vertebral") OR ("C7" ADJ6 "vertebral")).ti,ab) AND (exp "Spondylosis"/ OR "spondylosis".mp OR "Spondylolysis".mp OR "degenerative".mp OR "degenerat*".mp) AND ("sagittal alignment".mp OR "sagittal alignments".mp OR "sagittal balance".mp OR "sagittal balanced".mp OR "sagittal balances".mp OR "sagittal balancing".mp OR "sagittal profile".mp OR "sagittal parameters".mp OR "sagittal parameter".mp OR (("sagittal" ADJ22 "alignment") OR ("sagittal" ADJ22 "alignments") OR ("sagittal" ADJ22 "balance") OR ("sagittal" ADJ22 "imbalance") OR ("sagittal" ADJ22 "balanced") OR ("sagittal" ADJ22 "balances") OR ("sagittal" ADJ22 "balancing")).ti,ab OR ((exp "Postural Balance"/ OR "Posture Balance".mp OR "Posture Equilibrium".mp OR "Musculoskeletal Equilibrium".mp OR "Postural Equilibrium".mp OR "Postural Control".mp OR "Posture Control".mp) AND ("sagittal".mp OR "sagittal*".mp))) NOT (("Case Reports"/ OR "case report".ti OR "case rep".af) NOT (exp "Review"/ OR "review".ti OR exp "Clinical Study"/ OR "trial".ti OR "RCT".ti)) AND (english.la OR dutch.la))

**Embase**497 hits, 151 unique
((exp *"Cervical Vertebra"/ OR exp "Cervical Spine"/ OR "cervical spine".ti,ab OR "cervical spines".ti,ab OR "cervical spinal".ti,ab OR "Cervical Vertebrae".ti,ab OR "Cervical Vertebra".ti,ab OR "Cervical Atlas".ti,ab OR "odontoid process".ti,ab OR exp *"Cervical Spinal Cord"/ OR "Cervical Cord".ti,ab OR "cervical".ti,ab OR "cervical*".ti,ab OR (("C1" ADJ6 "spine") OR ("C2" ADJ6 "spine") OR ("C3" ADJ6 "spine") OR ("C4" ADJ6 "spine") OR ("C5" ADJ6 "spine") OR ("C6" ADJ6 "spine") OR ("C7" ADJ6 "spine") OR ("C1" ADJ6 "vertebra") OR ("C2" ADJ6 "vertebra") OR ("C3" ADJ6 "vertebra") OR ("C4" ADJ6 "vertebra") OR ("C5" ADJ6 "vertebra") OR ("C6" ADJ6 "vertebra") OR ("C7" ADJ6 "vertebra") OR ("C1" ADJ6 "vertebral") OR ("C2" ADJ6 "vertebral") OR ("C3" ADJ6 "vertebral") OR ("C4" ADJ6 "vertebral") OR ("C5" ADJ6 "vertebral") OR ("C6" ADJ6 "vertebral") OR ("C7" ADJ6 "vertebral")).ti,ab) AND (exp *"Spondylosis"/ OR "spondylosis".ti,ab OR "Spondylolysis".ti,ab OR "degenerative".ti,ab OR "degenerat*".ti,ab OR (*"Spine Disease"/ AND *"Degenerative Disease"/)) AND (exp "sagittal parameter"/ OR "sagittal alignment".ti,ab OR "sagittal alignments".ti,ab OR "sagittal balance".ti,ab OR "sagittal balanced".ti,ab OR "sagittal balances".ti,ab OR "sagittal balancing".ti,ab OR "sagittal profile".ti,ab OR "sagittal parameters".ti,ab OR "sagittal parameter".ti,ab OR (("sagittal" ADJ22 "alignment") OR ("sagittal" ADJ22 "alignments") OR ("sagittal" ADJ22 "balance") OR ("sagittal" ADJ22 "imbalance") OR ("sagittal" ADJ22 "balanced") OR ("sagittal" ADJ22 "balances") OR ("sagittal" ADJ22 "balancing")).ti,ab OR ((exp *"Body equilibrium"/ OR "Posture Balance".ti,ab OR "Posture Equilibrium".ti,ab OR "Musculoskeletal Equilibrium".ti,ab OR "Postural Equilibrium".ti,ab OR "Postural Control".ti,ab OR "Posture Control".ti,ab) AND ("sagittal".ti,ab OR "sagittal*".ti,ab))) NOT (("Case Report"/ OR "case report".ti OR (case AND (report OR reports)).jw) NOT (exp "Review"/ OR "review".ti OR "Clinical Study"/ OR exp "Clinical Trial"/ OR "trial".ti OR "RCT".ti)) AND (english.la OR dutch.la) NOT (conference review or conference abstract).pt)
 **Web of Science**375 hits, 24 unique
((TI=("Cervical Vertebra" OR "Cervical Spine" OR "cervical spine" OR "cervical spines" OR "cervical spinal" OR "Cervical Vertebrae" OR "Cervical Vertebra" OR "Cervical Atlas" OR "odontoid process" OR "Cervical Spinal Cord" OR "Cervical Cord" OR "cervical" OR "cervical*" OR (("C1" NEAR/6 "spine") OR ("C2" NEAR/6 "spine") OR ("C3" NEAR/6 "spine") OR ("C4" NEAR/6 "spine") OR ("C5" NEAR/6 "spine") OR ("C6" NEAR/6 "spine") OR ("C7" NEAR/6 "spine") OR ("C1" NEAR/6 "vertebra") OR ("C2" NEAR/6 "vertebra") OR ("C3" NEAR/6 "vertebra") OR ("C4" NEAR/6 "vertebra") OR ("C5" NEAR/6 "vertebra") OR ("C6" NEAR/6 "vertebra") OR ("C7" NEAR/6 "vertebra") OR ("C1" NEAR/6 "vertebral") OR ("C2" NEAR/6 "vertebral") OR ("C3" NEAR/6 "vertebral") OR ("C4" NEAR/6 "vertebral") OR ("C5" NEAR/6 "vertebral") OR ("C6" NEAR/6 "vertebral") OR ("C7" NEAR/6 "vertebral"))) OR AK=("Cervical Vertebra" OR "Cervical Spine" OR "cervical spine" OR "cervical spines" OR "cervical spinal" OR "Cervical Vertebrae" OR "Cervical Vertebra" OR "Cervical Atlas" OR "odontoid process" OR "Cervical Spinal Cord" OR "Cervical Cord" OR "cervical" OR "cervical*" OR (("C1" NEAR/6 "spine") OR ("C2" NEAR/6 "spine") OR ("C3" NEAR/6 "spine") OR ("C4" NEAR/6 "spine") OR ("C5" NEAR/6 "spine") OR ("C6" NEAR/6 "spine") OR ("C7" NEAR/6 "spine") OR ("C1" NEAR/6 "vertebra") OR ("C2" NEAR/6 "vertebra") OR ("C3" NEAR/6 "vertebra") OR ("C4" NEAR/6 "vertebra") OR ("C5" NEAR/6 "vertebra") OR ("C6" NEAR/6 "vertebra") OR ("C7" NEAR/6 "vertebra") OR ("C1" NEAR/6 "vertebral") OR ("C2" NEAR/6 "vertebral") OR ("C3" NEAR/6 "vertebral") OR ("C4" NEAR/6 "vertebral") OR ("C5" NEAR/6 "vertebral") OR ("C6" NEAR/6 "vertebral") OR ("C7" NEAR/6 "vertebral"))) OR AB=("Cervical Vertebra" OR "Cervical Spine" OR "cervical spine" OR "cervical spines" OR "cervical spinal" OR "Cervical Vertebrae" OR "Cervical Vertebra" OR "Cervical Atlas" OR "odontoid process" OR "Cervical Spinal Cord" OR "Cervical Cord" OR "cervical" OR "cervical*" OR (("C1" NEAR/6 "spine") OR ("C2" NEAR/6 "spine") OR ("C3" NEAR/6 "spine") OR ("C4" NEAR/6 "spine") OR ("C5" NEAR/6 "spine") OR ("C6" NEAR/6 "spine") OR ("C7" NEAR/6 "spine") OR ("C1" NEAR/6 "vertebra") OR ("C2" NEAR/6 "vertebra") OR ("C3" NEAR/6 "vertebra") OR ("C4" NEAR/6 "vertebra") OR ("C5" NEAR/6 "vertebra") OR ("C6" NEAR/6 "vertebra") OR ("C7" NEAR/6 "vertebra") OR ("C1" NEAR/6 "vertebral") OR ("C2" NEAR/6 "vertebral") OR ("C3" NEAR/6 "vertebral") OR ("C4" NEAR/6 "vertebral") OR ("C5" NEAR/6 "vertebral") OR ("C6" NEAR/6 "vertebral") OR ("C7" NEAR/6 "vertebral")))) AND (TI=("Spondylosis" OR "spondylosis" OR "Spondylolysis" OR "degenerative" OR "degenerat*") OR AK=("Spondylosis" OR "spondylosis" OR "Spondylolysis" OR "degenerative" OR "degenerat*") OR AB=("Spondylosis" OR "spondylosis" OR "Spondylolysis" OR "degenerative" OR "degenerat*")) AND (TI=("sagittal parameter" OR "sagittal alignment" OR "sagittal alignments" OR "sagittal balance" OR "sagittal balanced" OR "sagittal balances" OR "sagittal balancing" OR "sagittal profile" OR "sagittal parameters" OR "sagittal parameter" OR (("sagittal" NEAR/22 "alignment") OR ("sagittal" NEAR/22 "alignments") OR ("sagittal" NEAR/22 "balance") OR ("sagittal" NEAR/22 "imbalance") OR ("sagittal" NEAR/22 "balanced") OR ("sagittal" NEAR/22 "balances") OR ("sagittal" NEAR/22 "balancing")) OR (("Body equilibrium" OR "Posture Balance" OR "Posture Equilibrium" OR "Musculoskeletal Equilibrium" OR "Postural Equilibrium" OR "Postural Control" OR "Posture Control") AND ("sagittal" OR "sagittal*"))) OR AK=("sagittal parameter" OR "sagittal alignment" OR "sagittal alignments" OR "sagittal balance" OR "sagittal balanced" OR "sagittal balances" OR "sagittal balancing" OR "sagittal profile" OR "sagittal parameters" OR "sagittal parameter" OR (("sagittal" NEAR/22 "alignment") OR ("sagittal" NEAR/22 "alignments") OR ("sagittal" NEAR/22 "balance") OR ("sagittal" NEAR/22 "imbalance") OR ("sagittal" NEAR/22 "balanced") OR ("sagittal" NEAR/22 "balances") OR ("sagittal" NEAR/22 "balancing")) OR (("Body equilibrium" OR "Posture Balance" OR "Posture Equilibrium" OR "Musculoskeletal Equilibrium" OR "Postural Equilibrium" OR "Postural Control" OR "Posture Control") AND ("sagittal" OR "sagittal*"))) OR AB=("sagittal parameter" OR "sagittal alignment" OR "sagittal alignments" OR "sagittal balance" OR "sagittal balanced" OR "sagittal balances" OR "sagittal balancing" OR "sagittal profile" OR "sagittal parameters" OR "sagittal parameter" OR (("sagittal" NEAR/22 "alignment") OR ("sagittal" NEAR/22 "alignments") OR ("sagittal" NEAR/22 "balance") OR ("sagittal" NEAR/22 "imbalance") OR ("sagittal" NEAR/22 "balanced") OR ("sagittal" NEAR/22 "balances") OR ("sagittal" NEAR/22 "balancing")) OR (("Body equilibrium" OR "Posture Balance" OR "Posture Equilibrium" OR "Musculoskeletal Equilibrium" OR "Postural Equilibrium" OR "Postural Control" OR "Posture Control") AND ("sagittal" OR "sagittal*")))) NOT (TI=("Case Report") OR AK=("case report")) AND LA=(english OR dutch) NOT DT=(meeting abstract))

**Cochrane Library**23 hits, 12 unique
(("Cervical Vertebra" OR "Cervical Spine" OR "cervical spine" OR "cervical spines" OR "cervical spinal" OR "Cervical Vertebrae" OR "Cervical Vertebra" OR "Cervical Atlas" OR "odontoid process" OR "Cervical Spinal Cord" OR "Cervical Cord" OR "cervical" OR "cervical*" OR (("C1" NEAR/6 "spine") OR ("C2" NEAR/6 "spine") OR ("C3" NEAR/6 "spine") OR ("C4" NEAR/6 "spine") OR ("C5" NEAR/6 "spine") OR ("C6" NEAR/6 "spine") OR ("C7" NEAR/6 "spine") OR ("C1" NEAR/6 "vertebra") OR ("C2" NEAR/6 "vertebra") OR ("C3" NEAR/6 "vertebra") OR ("C4" NEAR/6 "vertebra") OR ("C5" NEAR/6 "vertebra") OR ("C6" NEAR/6 "vertebra") OR ("C7" NEAR/6 "vertebra") OR ("C1" NEAR/6 "vertebral") OR ("C2" NEAR/6 "vertebral") OR ("C3" NEAR/6 "vertebral") OR ("C4" NEAR/6 "vertebral") OR ("C5" NEAR/6 "vertebral") OR ("C6" NEAR/6 "vertebral") OR ("C7" NEAR/6 "vertebral"))) AND ("Spondylosis" OR "spondylosis" OR "Spondylolysis" OR "degenerative" OR "degenerat*") AND ("sagittal parameter" OR "sagittal alignment" OR "sagittal alignments" OR "sagittal balance" OR "sagittal balanced" OR "sagittal balances" OR "sagittal balancing" OR "sagittal profile" OR "sagittal parameters" OR "sagittal parameter" OR (("sagittal" NEAR/22 "alignment") OR ("sagittal" NEAR/22 "alignments") OR ("sagittal" NEAR/22 "balance") OR ("sagittal" NEAR/22 "imbalance") OR ("sagittal" NEAR/22 "balanced") OR ("sagittal" NEAR/22 "balances") OR ("sagittal" NEAR/22 "balancing")) OR (("Body equilibrium" OR "Posture Balance" OR "Posture Equilibrium" OR "Musculoskeletal Equilibrium" OR "Postural Equilibrium" OR "Postural Control" OR "Posture Control") AND ("sagittal" OR "sagittal*")))):ti,ab,kw **Emcare**104 hits, 8 unique
((exp *"Cervical Spine"/ OR "cervical spine".ti,ab OR "cervical spines".ti,ab OR "cervical spinal".ti,ab OR "Cervical Vertebrae".ti,ab OR "Cervical Vertebra".ti,ab OR "Cervical Atlas".ti,ab OR "odontoid process".ti,ab OR exp *"Cervical Spinal Cord"/ OR "Cervical Cord".ti,ab OR "cervical".ti,ab OR "cervical*".ti,ab OR (("C1" ADJ6 "spine") OR ("C2" ADJ6 "spine") OR ("C3" ADJ6 "spine") OR ("C4" ADJ6 "spine") OR ("C5" ADJ6 "spine") OR ("C6" ADJ6 "spine") OR ("C7" ADJ6 "spine") OR ("C1" ADJ6 "vertebra") OR ("C2" ADJ6 "vertebra") OR ("C3" ADJ6 "vertebra") OR ("C4" ADJ6 "vertebra") OR ("C5" ADJ6 "vertebra") OR ("C6" ADJ6 "vertebra") OR ("C7" ADJ6 "vertebra") OR ("C1" ADJ6 "vertebral") OR ("C2" ADJ6 "vertebral") OR ("C3" ADJ6 "vertebral") OR ("C4" ADJ6 "vertebral") OR ("C5" ADJ6 "vertebral") OR ("C6" ADJ6 "vertebral") OR ("C7" ADJ6 "vertebral")).ti,ab) AND (exp *"Spondylosis"/ OR "spondylosis".ti,ab OR exp *"Spondylolysis"/ OR "Spondylolysis".ti,ab OR "degenerative".ti,ab OR "degenerat*".ti,ab) AND ("sagittal alignment".ti,ab OR "sagittal alignments".ti,ab OR "sagittal balance".ti,ab OR "sagittal balanced".ti,ab OR "sagittal balances".ti,ab OR "sagittal balancing".ti,ab OR "sagittal profile".ti,ab OR "sagittal parameters".ti,ab OR "sagittal parameter".ti,ab OR (("sagittal" ADJ22 "alignment") OR ("sagittal" ADJ22 "alignments") OR ("sagittal" ADJ22 "balance") OR ("sagittal" ADJ22 "imbalance") OR ("sagittal" ADJ22 "balanced") OR ("sagittal" ADJ22 "balances") OR ("sagittal" ADJ22 "balancing")).ti,ab OR ((exp *"Body Equilibrium"/ OR "Posture Balance".ti,ab OR "Posture Equilibrium".ti,ab OR "Musculoskeletal Equilibrium".ti,ab OR "Postural Equilibrium".ti,ab OR "Postural Control".ti,ab OR "Posture Control".ti,ab) AND ("sagittal".ti,ab OR "sagittal*".ti,ab))) NOT (("Case Report"/ OR "case report".ti OR (case AND (report OR reports)).jw) NOT (exp "Review"/ OR "review".ti OR "Clinical Study"/ OR exp "Clinical Trial"/ OR "trial".ti OR "RCT".ti)) AND (english.la OR dutch.la)) NOT (("Case Reports"[ptyp] OR "case report"[ti] OR "case rep"[all fields]) NOT ("Review"[ptyp] OR "review"[ti] OR "Clinical Study"[ptyp] OR "trial"[ti] OR "RCT"[ti])) AND (english[la] OR dutch[la]))

**Academic Search Premier**209 hits, 8 unique
AND LA=(english OR dutch) NOT DT=(meeting abstract)
((TI("Cervical Vertebra" OR "Cervical Spine" OR "cervical spine" OR "cervical spines" OR "cervical spinal" OR "Cervical Vertebrae" OR "Cervical Vertebra" OR "Cervical Atlas" OR "odontoid process" OR "Cervical Spinal Cord" OR "Cervical Cord" OR "cervical" OR "cervical*" OR (("C1" NEAR/6 "spine") OR ("C2" NEAR/6 "spine") OR ("C3" NEAR/6 "spine") OR ("C4" NEAR/6 "spine") OR ("C5" NEAR/6 "spine") OR ("C6" NEAR/6 "spine") OR ("C7" NEAR/6 "spine") OR ("C1" NEAR/6 "vertebra") OR ("C2" NEAR/6 "vertebra") OR ("C3" NEAR/6 "vertebra") OR ("C4" NEAR/6 "vertebra") OR ("C5" NEAR/6 "vertebra") OR ("C6" NEAR/6 "vertebra") OR ("C7" NEAR/6 "vertebra") OR ("C1" NEAR/6 "vertebral") OR ("C2" NEAR/6 "vertebral") OR ("C3" NEAR/6 "vertebral") OR ("C4" NEAR/6 "vertebral") OR ("C5" NEAR/6 "vertebral") OR ("C6" NEAR/6 "vertebral") OR ("C7" NEAR/6 "vertebral"))) OR KW("Cervical Vertebra" OR "Cervical Spine" OR "cervical spine" OR "cervical spines" OR "cervical spinal" OR "Cervical Vertebrae" OR "Cervical Vertebra" OR "Cervical Atlas" OR "odontoid process" OR "Cervical Spinal Cord" OR "Cervical Cord" OR "cervical" OR "cervical*" OR (("C1" NEAR/6 "spine") OR ("C2" NEAR/6 "spine") OR ("C3" NEAR/6 "spine") OR ("C4" NEAR/6 "spine") OR ("C5" NEAR/6 "spine") OR ("C6" NEAR/6 "spine") OR ("C7" NEAR/6 "spine") OR ("C1" NEAR/6 "vertebra") OR ("C2" NEAR/6 "vertebra") OR ("C3" NEAR/6 "vertebra") OR ("C4" NEAR/6 "vertebra") OR ("C5" NEAR/6 "vertebra") OR ("C6" NEAR/6 "vertebra") OR ("C7" NEAR/6 "vertebra") OR ("C1" NEAR/6 "vertebral") OR ("C2" NEAR/6 "vertebral") OR ("C3" NEAR/6 "vertebral") OR ("C4" NEAR/6 "vertebral") OR ("C5" NEAR/6 "vertebral") OR ("C6" NEAR/6 "vertebral") OR ("C7" NEAR/6 "vertebral"))) OR AB("Cervical Vertebra" OR "Cervical Spine" OR "cervical spine" OR "cervical spines" OR "cervical spinal" OR "Cervical Vertebrae" OR "Cervical Vertebra" OR "Cervical Atlas" OR "odontoid process" OR "Cervical Spinal Cord" OR "Cervical Cord" OR "cervical" OR "cervical*" OR (("C1" NEAR/6 "spine") OR ("C2" NEAR/6 "spine") OR ("C3" NEAR/6 "spine") OR ("C4" NEAR/6 "spine") OR ("C5" NEAR/6 "spine") OR ("C6" NEAR/6 "spine") OR ("C7" NEAR/6 "spine") OR ("C1" NEAR/6 "vertebra") OR ("C2" NEAR/6 "vertebra") OR ("C3" NEAR/6 "vertebra") OR ("C4" NEAR/6 "vertebra") OR ("C5" NEAR/6 "vertebra") OR ("C6" NEAR/6 "vertebra") OR ("C7" NEAR/6 "vertebra") OR ("C1" NEAR/6 "vertebral") OR ("C2" NEAR/6 "vertebral") OR ("C3" NEAR/6 "vertebral") OR ("C4" NEAR/6 "vertebral") OR ("C5" NEAR/6 "vertebral") OR ("C6" NEAR/6 "vertebral") OR ("C7" NEAR/6 "vertebral")))) AND (TI("Spondylosis" OR "spondylosis" OR "Spondylolysis" OR "degenerative" OR "degenerat*") OR KW("Spondylosis" OR "spondylosis" OR "Spondylolysis" OR "degenerative" OR "degenerat*") OR AB("Spondylosis" OR "spondylosis" OR "Spondylolysis" OR "degenerative" OR "degenerat*")) AND (TI("sagittal parameter" OR "sagittal alignment" OR "sagittal alignments" OR "sagittal balance" OR "sagittal balanced" OR "sagittal balances" OR "sagittal balancing" OR "sagittal profile" OR "sagittal parameters" OR "sagittal parameter" OR (("sagittal" NEAR/22 "alignment") OR ("sagittal" NEAR/22 "alignments") OR ("sagittal" NEAR/22 "balance") OR ("sagittal" NEAR/22 "imbalance") OR ("sagittal" NEAR/22 "balanced") OR ("sagittal" NEAR/22 "balances") OR ("sagittal" NEAR/22 "balancing")) OR (("Body equilibrium" OR "Posture Balance" OR "Posture Equilibrium" OR "Musculoskeletal Equilibrium" OR "Postural Equilibrium" OR "Postural Control" OR "Posture Control") AND ("sagittal" OR "sagittal*"))) OR KW("sagittal parameter" OR "sagittal alignment" OR "sagittal alignments" OR "sagittal balance" OR "sagittal balanced" OR "sagittal balances" OR "sagittal balancing" OR "sagittal profile" OR "sagittal parameters" OR "sagittal parameter" OR (("sagittal" NEAR/22 "alignment") OR ("sagittal" NEAR/22 "alignments") OR ("sagittal" NEAR/22 "balance") OR ("sagittal" NEAR/22 "imbalance") OR ("sagittal" NEAR/22 "balanced") OR ("sagittal" NEAR/22 "balances") OR ("sagittal" NEAR/22 "balancing")) OR (("Body equilibrium" OR "Posture Balance" OR "Posture Equilibrium" OR "Musculoskeletal Equilibrium" OR "Postural Equilibrium" OR "Postural Control" OR "Posture Control") AND ("sagittal" OR "sagittal*"))) OR AB("sagittal parameter" OR "sagittal alignment" OR "sagittal alignments" OR "sagittal balance" OR "sagittal balanced" OR "sagittal balances" OR "sagittal balancing" OR "sagittal profile" OR "sagittal parameters" OR "sagittal parameter" OR (("sagittal" NEAR/22 "alignment") OR ("sagittal" NEAR/22 "alignments") OR ("sagittal" NEAR/22 "balance") OR ("sagittal" NEAR/22 "imbalance") OR ("sagittal" NEAR/22 "balanced") OR ("sagittal" NEAR/22 "balances") OR ("sagittal" NEAR/22 "balancing")) OR (("Body equilibrium" OR "Posture Balance" OR "Posture Equilibrium" OR "Musculoskeletal Equilibrium" OR "Postural Equilibrium" OR "Postural Control" OR "Posture Control") AND ("sagittal" OR "sagittal*")))) NOT (TI("Case Report") OR KW("case report")))
