# Supplementary material for: The association between cervical sagittal balance parameters and clinical outcomes after single-level surgery for cervical radiculopathy and/or stenosis: A systematic review and meta-analysis
Source: Brain Spine. 2026 Mar 24;6:106009. doi: 10.1016/j.bas.2026.106009 (PMC13087681; doi:10.1016/j.bas.2026.106009)
Supplement: Multimedia component 2 [file mmc2.docx]

**Appendix B**: Quality assessment checklist according to an adjusted version of the Dutch Cochrane Centre checklist.

| **Section** | **Award 1 point if** |
| --- | --- |
| **Selection bias (3 pts.)** |  |
| Goal and inclusion | Goal of the study is stated and study explicitly states the inclusion criteria; |
| Selection of patients | Selective recruitment of patients can be ruled out; |
| Patient characteristics | Study reports the age range and mean age and states the distribution of gender; |
| **Outcome bias (3 pts.)** |  |
| Definition of Sagittal Balance | Definition of sagittal balance parameters, classification and radiological tools to measure sagittal balance were stated; |
| Clinical outcome | Clinical outcome was systematically evaluated in correlation to sagittal balance; |
| Measurement | Sagittal Balance was measured on an X-Ray |
| **Follow-up bias (3 pts.)** |  |
| Correlation | Baseline Sagittal Balance with Baseline Clinical Outcome |
| Follow-up time frame | In Case of Follow-up: Follow-up range, period and mean were given and loss to follow-up<20%; |
| Multiple moments | Correlation through multiple time moments |
| **Total (9 pts.)** |  |
